# Editorial: Dynamics and functional exploration of pharmacologically active proteins

**DOI:** 10.3389/fchem.2026.1878522

**Published:** 2026-06-03

**Authors:** Zhenlu Li, Camelia Bala, Liqun Zhang

**Affiliations:** 1 Tianjin University, Tianjin, China; 2 Department of Analytical Chemistry and Physical Chemistry, University of Bucharest, Bucharest, Romania; 3 Hampton University, Hampton, VA, United States

**Keywords:** computational biology, drug discovery, modeling, molecular dynamics simulation, protein

Pharmacologically active proteins are proteins in the body that interact with drugs, therapeutic compounds, or biologically active molecules to produce a physiological effect (Leader et al., 2008). They play an important role in modern biomedical research and drug discovery, for example, by regulating critical physiological processes and serving as major therapeutic targets. They can be classified into enzymes, receptors, ion channels, transporters, and signaling molecules. Protein dynamics refers to the conformational flexibility and movement of proteins under physiological conditions (Kleckner and Foster, 2011). Unlike static structural models obtained through traditional crystallography, proteins exist in multiple conformational states that influence ligand binding, catalytic activity, and intermolecular interactions. Advancements in structural biology techniques such as cryo-electron microscopy (cryo-EM), nuclear magnetic resonance (NMR) spectroscopy, and molecular dynamics (MD) simulations have significantly improved our ability to study these dynamic behaviors in real time (Zadorozhnyi et al., 2024). Protein dynamics is tightly connected to its unique function (OrozcoM, 2014), and biomolecular dynamic properties underlie biological function (Bahar et al., 2010). Protein dynamics and molecular interactions play fundamental roles in regulating biological processes associated with cancer, immunity, inflammation, and neurodegenerative diseases (Penfield and Zhang, 2024; Kolch et al., 2015; Kulkarni and Kulkarni, 2019; Lin et al., 2025; Mercier and LaPointe, 2022; Seo and Park, 2020). Understanding protein structural dynamics and functional mechanisms has become essential for the development of effective and selective drugs. Recent advances in computational simulations, artificial intelligence (AI), and experimental biophysics have significantly improved our ability to investigate protein structure–function relationships and accelerate therapeutic discovery (Frasnetti et al., 2024; Zhang et al., 2024).

The Research Topic reflects several important emerging directions in modern computational biology and drug discovery. The studies collectively demonstrate how molecular docking, molecular dynamics (MD) simulations, machine learning, pharmacophore modeling prediction can be integrated to investigate disease-associated proteins and identify promising therapeutic compounds.

Xu et al. identified HCJ007 as a potent squalene epoxidase (SQLE) inhibitor for pancreatic cancer therapy using an active learning-assisted screening strategy. The team used Schrödinger’s DeepAutoQSAR to iteratively train machine learning models based on docking scores and binding affinities. After three rounds of refinement, top candidates from a marine natural product library were validated through MD simulations and ADMET analysis. This study demonstrates how AI-driven pipelines significantly improve screening efficiency. It also highlights the value of marine natural products as a vital source for new anticancer drugs.

In another contribution, Al Khzem and Ahmad targeted haptoglobin (PDB: 4X0L) to develop treatments for inflammation and hematological malignancies. By screening the DrugBank database with XP docking and MM/GBSA calculations, they identified five promising inhibitors, including L-histidinol phosphate and L-gluconic acid. The study utilized 100 ns molecular dynamics simulations and WaterMap analysis to confirm strong binding stability. Furthermore, DFT computations and pharmacokinetic profiling validated the drug-like potential of these compounds. This integrative workflow highlights the efficiency of computational chemistry in discovering new leads for cancer-associated pathological processes.

Hayek-Orduz et al. developed dyphAI, an ensemble platform integrating machine learning with dynamic pharmacophore modeling and MD simulations to identify novel acetylcholinesterase (AChE) inhibitors for Alzheimer’s disease. The pipeline utilized RDKit descriptors and Optuna-optimized models to screen the ZINC database, pinpointing 18 candidates with high predicted binding energies targeting key residues like Trp-86. While the computational approach successfully captured complex π-cation and π-π interactions, experimental validation of nine selected molecules yielded mixed results: only two candidates (4 and 7) matched or outperformed the control drug galantamine, while others showed varied inhibition or faced solubility Research Topic. Ultimately, the study highlights how incorporating protein flexibility into AI-driven workflows can enhance virtual screening reliability, though it also underscores the ongoing challenge of translating high-scoring computational hits into consistent experimental leads.

The Research Topic reflects the growing convergence of machine learning and molecular simulations in drug discovery. By combining multiple computational approaches, researchers can improve screening accuracy, accelerate lead optimization, and reduce the cost and time associated with early-stage therapeutic development. While experimental validation remains essential, these studies demonstrate the substantial contribution of computational methodologies to identifying promising therapeutic candidates and understanding complex biological mechanisms.
